# Ga···C Triel Bonds—Why They Are Not Strong Enough to Change Trigonal Configuration into Tetrahedral One: DFT Calculations on Dimers That Occur in Crystal Structures

**DOI:** 10.3390/ijms241512212

**Published:** 2023-07-30

**Authors:** Sławomir J. Grabowski

**Affiliations:** 1 Polimero eta Material Aurreratuak: Fisika, Kimika eta Teknologia, Kimika Fakultatea, Euskal Herriko Unibertsitatea UPV/EHU & Donostia International Physics Center (DIPC) PK 1072, 20080 Donostia, Spain; s.grabowski@ikerbasque.org; 2 Ikerbasque, Basque Foundation for Science, 48011 Bilbao, Spain

**Keywords:** Ga···C triel bond, crystal structures, quantum theory of atoms in molecules, natural bond orbital, energy decomposition analysis

## Abstract

Structures characterized by the trigonal coordination of the gallium center that interacts with electron rich carbon sites are described. These interactions may be classified as Ga···C triel bonds. Their properties are analyzed in this study since these interactions may be important in numerous chemical processes including catalytical activities; additionally, geometrical parameters of corresponding species are described. The Ga···C triel bonds discussed here, categorized also as the π-hole bonds, do not change the trigonal configuration of the gallium center into the tetrahedral one despite total interactions in dimers being strong; however, the main contribution to the stabilization of corresponding structures comes from the electrostatic forces. The systems analyzed theoretically here come from crystal structures since the Cambridge Structural Database, CSD, search was performed to find structures where the gallium center linked to CC bonds of Lewis base units occurs. The majority structures found in CSD are characterized by parallel, stacking-like arrangements of species containing the Ga-centers. The theoretical results show that interactions within dimers are not classified as the three-centers links as in a case of typical hydrogen bonds and numerous other interactions. The total interactions in dimers analyzed here consist of several local intermolecular atom–atom interactions; these are mainly the Ga···C links. The DFT results are supported in this study by calculations with the use of the quantum theory of atoms in molecules, QTAIM, the natural bond orbital, NBO, and the energy decomposition analysis, EDA, approaches.

## 1. Introduction

The hydrogen bond seems to be one of the most important interactions that steers arrangements of molecules and ions in crystals. Not only in crystals, its role in liquids and in a gas phase is also often analyzed and discussed using experimental and theoretical approaches [1,2,3,4,5,6,7,8]. However, there are other interactions that play an important role in numerous processes and chemical reactions [9,10,11,12,13,14,15,16,17,18]. The majority of them are classified as σ-hole bonds or π-hole bonds [12,15,16,17,18]. According to the concept introduced by Murray, Politzer, and coworkers [19,20,21,22], in these interactions, the centers of Lewis acid units are characterized by the depletion of the electron charge and if this depletion is sufficient, it leads to the positive electrostatic potential, EP, in areas of these centers. If areas of the electron charge depletion occur in elongations of bonds to these centers, then they are named as σ-holes [19,20,21,22] while if the electron charge depletions are observed in areas that are perpendicular to planar molecular fragments, then they are named as π-holes [15,16].

Special attention has been paid in numerous studies on triel bonds that are formed between elements of the 13th group (triel elements) acting as electrophiles and the electron-rich sites of Lewis base units [23,24]. The triel trivalent centers possess six electrons in the valence shell, thus they do not obey the octet rule [25]; such centers are known as hypovalent in contrast to the hypervalent centers that contain more than eight electrons in the valence shell and thus also do not obey this rule [26]. This is why the triel centers are characterized by strong electrophilic properties, since interacting with electron donating ligands, they may fulfill the octet rule. However, it was found that the triel centers differ significantly if the individual elements are considered [23]. For example, in the case of complexes of aluminum trihalides, the intermolecular links to aluminum centers are mostly electrostatic interactions while in the remaining complexes of triel trihalides, the connections to triel centers possess characteristics of partly covalent interactions [23]. Moreover, it was found that an interaction of planar triel trivalent species with one ligand leads, in a case of a strong interaction, to a change of the trigonal planar configuration into the tetrahedral configuration [23,24]. The interaction of such tetrahedral triel species with the next ligand leads to the next change of the configuration into the trigonal bipyramid [23,27]. Such changes of configurations are related to types of interactions, to strengths of the latter ones, and to the valence shell–electron pair repulsion model [27,28].

There are numerous studies related to structures and properties of boron and aluminum compounds that are very important in chemical reactions and processes including catalysis [29,30,31,32,33]. For example, boron plays a crucial role in hydrogen storage [30,31,32]. Thus, experimental and theoretical studies concerning interactions of B and Al species have been performed; for example, the planar trivalent species of these elements interact with nucleophiles through π-holes; next, it seems that tetravalent species interact with electron-rich species through σ-holes [23,24,27].

In this study, the species containing the gallium atom that acts as the Lewis acid center through the π-hole area are analyzed since studies concerning interaction of heavier triel elements are not as numerous as in a case of lighter boron and aluminum. However, it is worth noting that articles, particularly those concerning configurations of heavier triel species, appear from time to time [34]. The interactions of the gallium trivalent center that possesses trigonal configuration are analyzed here. The π-electron systems were chosen as the Lewis base units since they may act as nucleophiles through CC unsaturated bonds or through the electron-rich carbon centers. It is worth noting that such interactions may be important in numerous chemical processes as was indicated in several earlier studies. For example, several studies may be mentioned: the early study on the gallium bromide catalyzed alkylation of aromatics with ethyl bromide [35], the analysis of the role of gallium complexes for the catalytic hydroboration of aldehydes, ketones, and carbon dioxide [36], the role of elemental gallium center for catalytic formation of the carbon–carbon bonds [37], or the anticancer properties of gallium–chrisin complexes [38]. A recent study that is not related to CC bonds, but to analogues nitrogen systems, i.e., electrocatalytic activity of Ga towards the nitrogen reduction reaction, may be mentioned here [39].

One can see that the gallium–carbon and gallium π-electrons interactions play an important role in processes mentioned above. Hence, it is analyzed in this study if interactions of gallium centers with weak electron donors such as carbon centers may lead to a change of the gallium configuration from trigonal to tetrahedral configuration. The analysis of properties of such interactions may be important for understanding numerous reactions and processes. This is why in the first step of this study, the corresponding structures found in Cambridge Structural Database, CSD [40,41], that contain the Ga···π (or Ga···C) contacts were described and discussed. Next, for the selected structures taken from CSD, the high-level density functional theory, DFT, calculations were performed to deepen the understanding of nature of these interactions. The above-mentioned structures found in CSD contain planar aromatic systems (this is described later here) most often and the Ga-centers attached. These structures are discussed here since the majority of them are characterized by arrangements similar to the stacking ones that are often analyzed in other studies.

## 2. Results and Discussion

### 2.1. Ga···C Triel Bonds in Crystal Structures

A search through the Cambridge Structural Database, CSD [40,41] (November 2022 release), was performed here to find crystal structures containing species with the gallium trivalent center that interacts with the carbon system acting as the Lewis base site. The following criteria for this search were applied: 3D coordinates determined, only no disordered structures, no errors, no polymeric structures, R-factor less or equal to 7.5%, and only single crystal structures. The electron donor is defined as follows: the Ga-center is in contact with two carbon atoms that form a covalent bond. The distances between the gallium center and such carbon centers are shorter than the sum of the corresponding C and Ga van der Waals radii. The radii proposed by Bondi [42] which are inserted in CSD were applied here. The choice of systems where Ga-center is attached to two carbon sites increases the possibility of finding the rich electron structures. For example, for the Z···π triel bonds in ZX_3_···C_2_H_2_ and ZX_3_···C_2_H_4_ complexes (Z = B, Al and X = H, F, Cl, Br), the Z···C contacts of equal lengths (or nearly so) are observed for each complex [43]. This occurs because of interactions of electrophilic Z-centers with π-electrons of CC bonds. Hence, the aim of the search performed here was to find complexes with π-electrons or electron rich carbon atoms that play a role of Lewis bases centers.

Thirteen crystal structures were found in CSD that fulfil the search criteria presented above. The CC bond length of carbon centers in the shortest contacts with the Ga-center is in the 1.346 Å–1.446 Å range; this length amounts 1.515 Å in only one case. This may suggest that the π-electrons of non-saturated CC bonds are important in Ga···π triel bonds that may occur here. However, this is in contrast to the observed Ga··CC (bond) distances; in all cases one of two Ga···C contacts is significantly shorter (at least by ~0.3–0.4 Å) than the other one. Only in two crystal structures (NIZZUS and QATMUT refcodes, see Table 1), the pairs of Ga···C contacts with CC bonds are characterized by distances of lengths differing by less than 0.2 Å. Hence, one may expect the occurrence of Ga···C triel bonds rather, not Ga···π interactions.

Table 1 presents crystal structures that were found in CSD; i.e., names of compounds, refcodes of crystal structures and Ga···C distances (the shortest distance for each crystal structure is shown). The shortest Ga···C distances from BP86-D4/TZ2P calculations (descriptions of theoretical methods are in the section Materials and Methods) on dimers corresponding to their analogues in crystals are also included in this table. Only complexes from nine crystal structures were theoretically analyzed further here since four other structures are characterized by greater complexity. For example, for the UJIXEQ structure, ferrocene fragments are observed, which increases the complexity of the system that could be considered. Furthermore, the majority of the remaining nine structures that are further discussed are characterized by similar arrangements where neighboring molecules are approximately parallel to each other, these are arrangements similar to the stacking ones. Table 1 contains also references to studies on crystal structures that are the result of the CSD search.

The experimental Ga···C distances are usually longer that those resulting from calculations, the reverse situation is observed only in two structures (DMGACP and LEBNAH refcodes), i.e., the distances taken from crystal structures are shorter than those derived from calculations. The latter situation is more common since calculations concern two neighboring species while an occurrence of numerous species and forces in crystals is usually responsible for the “short-circuiting” of units that form the crystal structure. It is often explained as an effect of the packing forces [54]. This is the case in only two out of nine structures analyzed here.

Let us discuss arrangements in nine structures included in Table 1 which are also analyzed theoretically. In seven structures, arrangements similar to stacking ones occur; they are named here as stacking-like arrangements. In two cases (DMGACP and OFUSAJ), there are arrangements of other types. 

**Figure 1:**
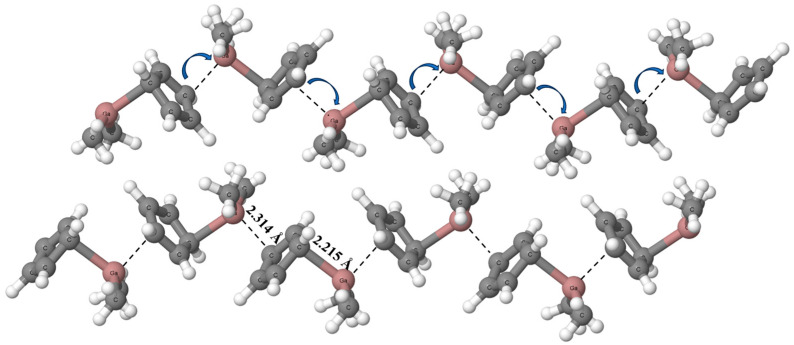
The fragment of the dimethyl(cyclopentadienyl)gallium crystal structure (DMGACP) [45]. The shortest Ga···C contacts are indicated by the broken lines. Arrows show the direction of the electron charge transfer.

Figure 1 presents the fragment of the dimethyl(cyclopentadienyl)gallium crystal structure (DMGACP). This structure is characterized by the P2_1_/c space group and four molecules in the unit cell (Z = 4). Hence, all molecules are equivalent by symmetry. In the neutral dimethyl(cyclopentadienyl)gallium unit, (CH_3_)_2_GaC_5_H_5_, the gallium center is trivalent. It is connected with two methyl groups and with the cyclopentadienyl anion. The latter connection may be treated formally as the Ga-C bond, its length amounts to 2.216 Å. There is another Ga···C link that may be treated as the intermolecular contact characterized by a length of 2.314 Å. The (CH_3_)_2_GaC_5_H_5_ species acts here simultaneously as the Lewis acid and Lewis base unit, through the gallium center and through the cyclopentadienyl ring, respectively. Hence, one may expect cooperativity effects by the Ga···C triel bonds. Fig. 1 shows arrangements of molecules in this crystal structure where “chains” of the (CH_3_)_2_GaC_5_H_5_ units occur. For one of such chains in Figure 1, the arrows indicate the direction of the electron charge transfer. Due to translational symmetry, the same gallium–carbon pairs of 2.216 Å (bond) and of 2.314 Å (intermolecular contact) occur alternatively in chains of molecules presented here.

In the case of the crystal structure of dimethyl (cyclopentadienyl) gallium, very short Ga···C contacts are observed (Figure 1) that may be a result of packing forces; however, the electron charge shifts in this structure suggest the occurrence of a cooperativity effect. The chains of molecules are observed in this structure where equivalent units act as Lewis bases and Lewis acids simultaneously. One can see that this cooperativity occurs for the same kind of interactions—triel bonds. There are other studies where cooperativity of the triel bond with other interactions occur [55,56,57,58,59,60,61,62,63,64]. However, there are probably no studies where only the coexistence of triel bonds enhances these interactions.

**Figure 2:**
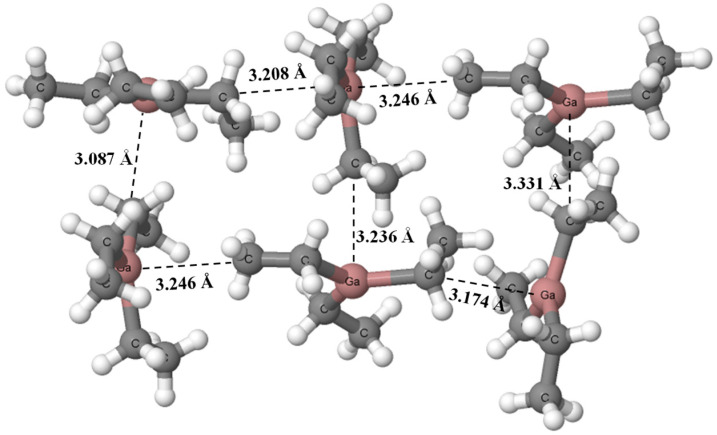
The fragment of the triethyl–gallium crystal structure (OFUSAJ) [51]. The shortest Ga···C contacts are indicated by the broken lines.

Figure 2 presents the fragment of the crystal structure of triethyl–gallium, another structure where parallel arrangements of molecules are not observed. The shortest Ga···C distances are close to the sum of corresponding gallium and carbon van der Waals radii. Hence, one cannot expect strong interactions here. The DFT results of calculations (Table 1) show the Ga···C theoretical distances shorter than experimental ones but still not so short.

**Figure 3:**
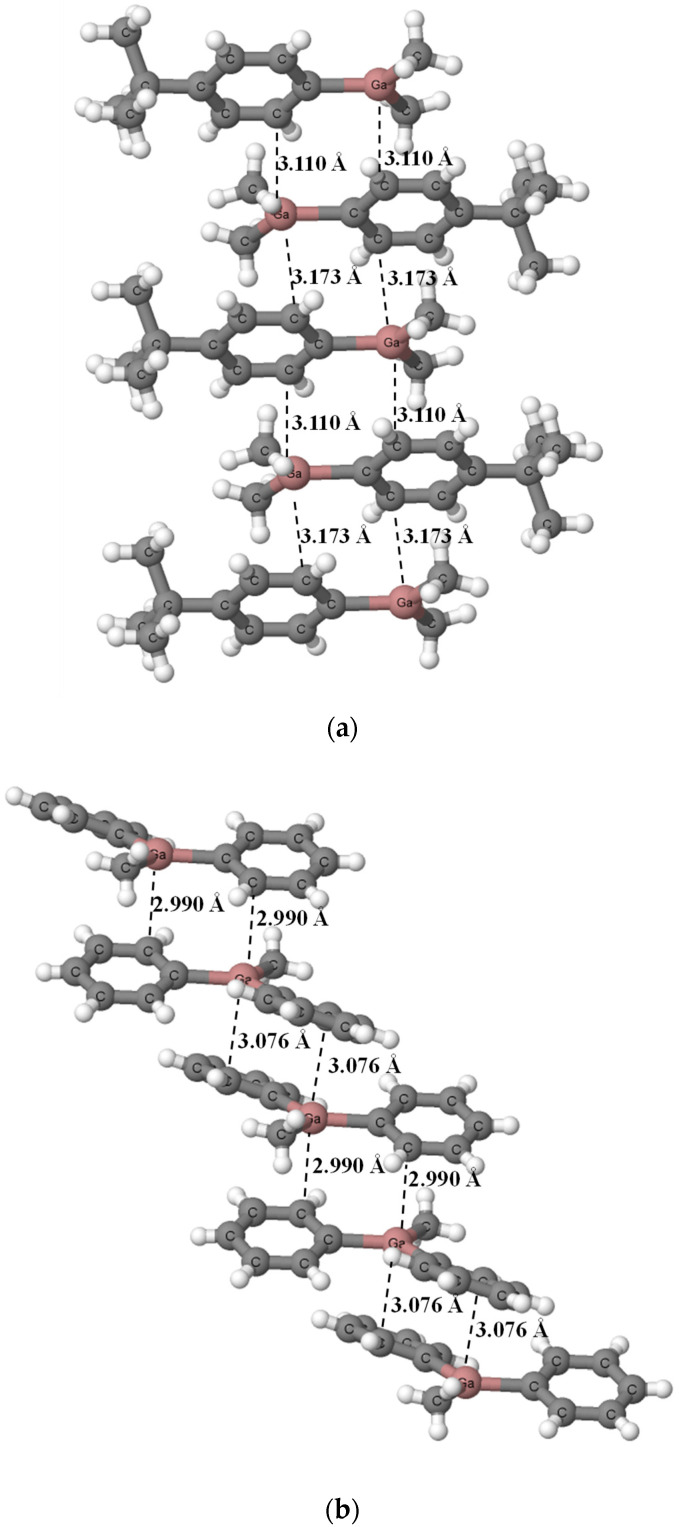
The fragments of crystal structures, (**a**) dimethyl-(4-t-butylphenyl)-gallium (HOJCEP) [46] and (**b**) methyl-diphenyl-gallium (HOJCIT) [46]. The shortest Ga···C contacts are indicated by the broken lines.

The remaining crystal structures that are also analyzed theoretically are characterized by stacking-like arrangements. Figure 3 presents two examples of such crystal structures, dimethyl-(4-t-butylphenyl)-gallium (HOJCEP) [46] and methyl-diphenyl-gallium (HOJCIT) [46].

One can see that layers of molecules in structures presented in Figure 3 are not regular, meaning that the Ga···C distances between “parallel” molecules are not regularly the same. For example, in the HOJCEP structure, the distances of 3.110 Å and 3.173 Å for the Ga···C contacts between layers are observed alternately. For the HOJCIT structure these are the distances equal to 2.990 Å and 3.078 Å, alternately. Similar situations are observed for other structures. The rough insight into the crystal structures containing such stacking-like arrangements shows that the gallium centers are characterized by the trigonal coordinations rather, in spite of their additional interactions with other layers, i.e., with carbon sites of other units containing Ga-centers. The planar trigonal configurations of Ga-centers are only slightly disturbed here.

One can see that the arrangements that occur in structures analyzed in this study are often referred to as the stacking ones. They are a subject of experimental studies and theoretical analyses where different interaction energy terms are discussed [65]. They are often named pi-stacking or pi–pi-stacking interactions. The role of dispersion forces has been analyzed for such arrangements. For example, 1,8-bis(phenylethynyl)anthracene was investigated experimentally in the gas phase and in the crystal structure; the additional DFT calculations were also performed there [66]. The role of π–π and σ(C-H) –π interactions was analyzed [66]. It is worth mentioning here that the role of displaced configurations is often undertaken [65,66]. Such displaced arrangements are energetically superior to parallel (sandwich) arrangements where the greater importance of repulsive forces is detected.

### 2.2. Interaction Energies—EDA Analysis

**Figure 4:**
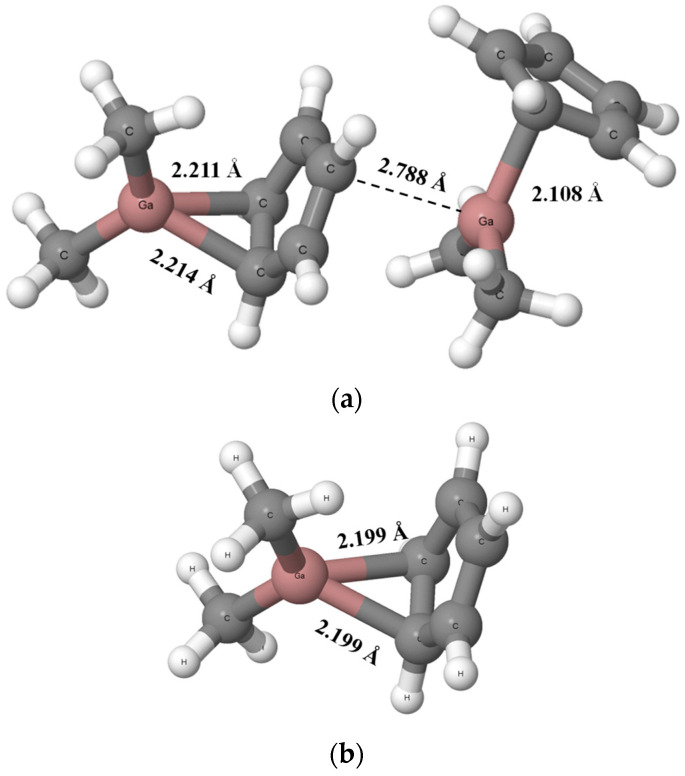
The dimer (**a**) and monomer (**b**) structures of dimethyl(cyclopentadienyl)gallium that result from the BP86-D4/TZ2P calculations. The shortest Ga···C contacts are indicated by the broken lines.

Figure 4 presents dimer and monomer structures of dimethyl (cyclopentadienyl) gallium that result from the BP86-D4/TZ2P calculations. One can see that one of molecules in the dimer acts as the Lewis acid and another one as the Lewis base (the right and the left molecules presented in Figure 4a, respectively). For this dimer, the Ga-C bond length for the Lewis acid unit amounts to 2.108 Å while two Ga-C bonds of the Lewis base unit are equal to 2.214 Å and 2.211 Å. The Ga-C bond length in the dimethyl (cyclopentadienyl) gallium unit in crystal structure is equal to 2.216 Å. The Ga···C intermolecular distance in the calculated dimer is equal to 2.788 Å, which is much longer than 2.314 Å for the corresponding distance in the crystal structure. The Ga-C bond lengths in the calculated monomer are equal to 2.199 Å (Figure 4b).

The reasons for differences in DMGACP structure between experimental results (Figure 1) and theoretical BP86-D4/TZ2P calculations (Figure 4) are as follows: the experimental results concern the crystal structure (solid state) while calculations correspond to the gas phase, crystal packings [54] mentioned above also play an important role.

Let us discuss differences between monomeric and dimeric structures of dimethyl(cyclopentadienyl)gallium. Two equivalent Ga-C bonds are observed in monomer structure; in the dimer, the Lewis base unit gives up the electron charge to the second unit. The latter results in slight elongations of Ga-C bonds in the electron donor species, probably because of the outflow of the electron charge from the cyclopentadienyl and in consequence its weaker interaction with the gallium center. On the other hand, the Lewis acid unit receives electron charge which is further shifted in excess to the cyclopentadienyl fragment. This results in the greater positive charge of the gallium center and the greater negative charge of the cyclopentadienyl fragment in this unit in comparison with the monomer. The NBO Ga-charge for this center is equal to +1.258 au and +1.285 au for monomer and in the Lewis acid unit in dimer, respectively. This is the electron charge redistribution process similar to that occurring in the hydrogen bonded systems [26]. Finally, the single Ga-C bond is formed in the Lewis acid unit that is shorter than such bonds in the monomer.

Table 2 displays the interaction energy of nine dimers optimized here at the BP86-D4/TZ2P level. These systems are linked by strong and very strong interactions since the absolute values of interaction energies, |ΔE_int_|s, are situated in the 11.8–58.3 kcal/mol range. The weakest interaction occurs for the calculated dimer related to the OFUSAJ crystal structure that was discussed in the previous section as a structure where the longest Ga···C distances which correspond to stabilizing interactions are observed. The |ΔE_int_| is equal to 11.8 kcal/mol here. This is a unique calculated system where the electrostatic term is not the most important attractive interaction. Both the orbital and dispersion terms are more important for this system. The similar situation of weaker interaction occurs for the DMGACP structure where |ΔE_int_| is 11.9 kcal/mol. However, the electrostatic interaction is the most important attractive contribution for this system. For the corresponding crystal structure, the chains of dimethyl(cyclopentadienyl) gallium are observed. The cooperative effect enhances the triel bonds that link molecules. It was pointed out earlier here that the arrangements in the corresponding OFUSAJ and DMGACP crystal structures are not classified as stacking-like ones.

For other systems presented in Table 2, for the corresponding crystal structures, the stacking-like arrangements are observed. In all these cases the electrostatic term, ΔE_elstat_, is the most important attractive one. The orbital and dispersion terms, ΔE_orb_ and ΔE_disp_, respectively, are usually comparable to each other. Even the absolute value of dispersion term outweighs the orbital one sometimes; this occurs for the HOJCAL and HUGVIP structures.

**Figure 5:**
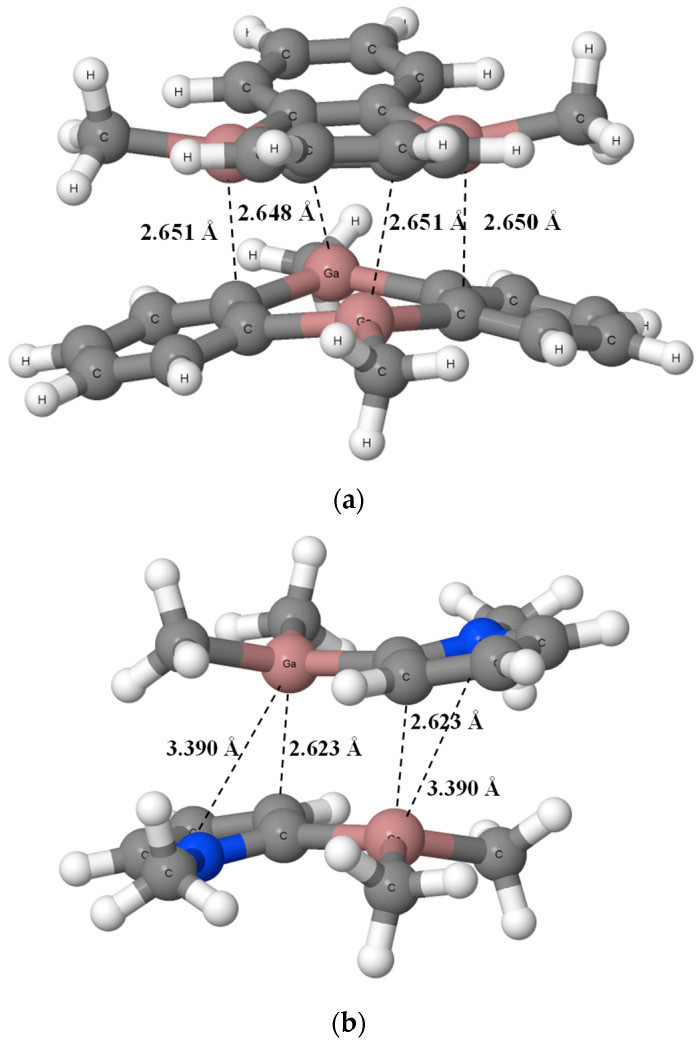
The structures of dimers calculated at the BP86-D4/TZ2P level, (**a**) 9,10-Dimethyl-9,10-dihydro-9,10-digalla-anthracene (NAZFIE), (**b**) Dimethyl- (N-methylpyrrol-2-yl)-gallium (LEBNAH). The shortest Ga···C and Ga···N contacts are indicated by the broken lines, nitrogen centers are designated by blue color.

For the majority of systems considered in this study the electrostatic interaction is the most important term, although other terms are not negligible and they are often comparable with the electrostatic contribution—one can see that from results of Table 2. Moreover, it seems that the greatest contributions to the energies of interactions come from the contacts between the gallium centers and carbon ones. The former centers are characterized by hypovalency and in consequence, the positive charge and positive electrostatic potential, while the carbon centers, often being components of aromatic systems, are characterized by the excess of the electron charge; in consequence, they act as the Lewis base centers. These explanations are confirmed by results of Table 2. The strongest interactions are observed for calculated dimers corresponding to the NAZFIE and LEBNAH crystal structures (Figure 5). For these structures, the percentage electrostatic interaction energy contribution to the total attractive interaction energy, (ΔE_elstat_/(ΔE_elstat_ + ΔE_elstat_ + ΔE_elstat_)) × 100%, is the greatest one, 44.1% and 46.5%, respectively. Furthermore, in the case of NAZFIE structure, there are two Ga-centers per single unit (monomer), which guarantees more Ga···C contacts that are attractive in dimer. 

Figure 5a presents the NAZFIE dimer where four such shortest attractive contacts are indicated. However, there are other Ga···C contacts shorter than 3 Å in this dimer which are important to stabilize this structure. In the case of LEBNAH structure, there is an additional nitrogen center per monomer. The nitrogen is a stronger Lewis base site than carbon center. Therefore, the occurrence of N-centers guarantees important additional local attractive interactions. Figure 5b presents the LEBNAH dimer where the Ga···C and Ga···N shortest contacts are indicated.

**Figure 6:**
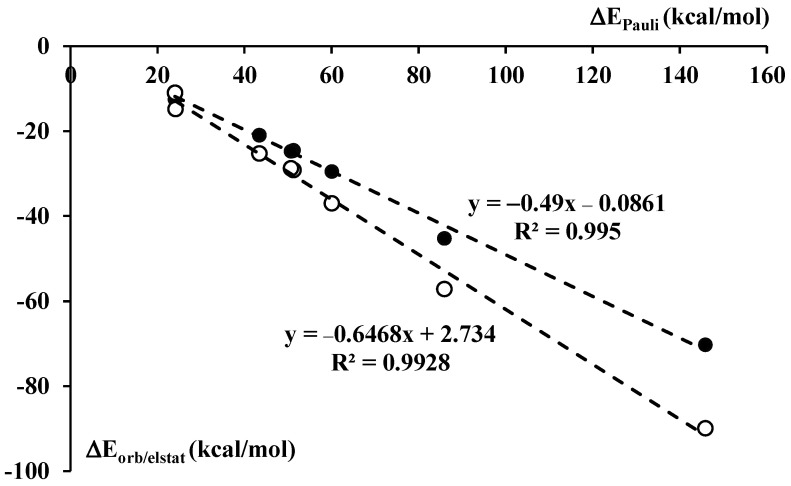
The linear correlations between the Pauli repulsion interaction energy, ΔE_Pauli_, and the attractive interaction energy terms, electrostatic contribution, ΔE_elstat_ (white circles), and the orbital energy contribution, ΔE_orb_ (black circles). The functions corresponding to these linear relationships and the linear correlation coefficients for these correlations are presented.

It was pointed out in former studies [67] that all interaction energy terms increase (their absolute values) with the increase of the strength of interaction. Such increases of attractive interaction energy terms (electrostatic, orbital, and dispersion) may also be treated as responses for the increase of the Pauli repulsion [67]. Figure 6 presents linear correlations between the Pauli repulsion and two attractive terms, electrostatic interaction energy and orbital interaction energy. The last term is related to the electron charge shifts resulting from the complex formation. These are shifts between the linked monomers and within them, often separated in other decomposition schemes into the charge transfer and polarization terms, respectively. One can see (Figure 6) excellent linear correlations. However, the correlation between the repulsion term and the dispersion interaction energy is poor since linear correlation coefficient low (R^2^ = 0.812).

### 2.3. QTAIM Parameters

**Figure 7:**
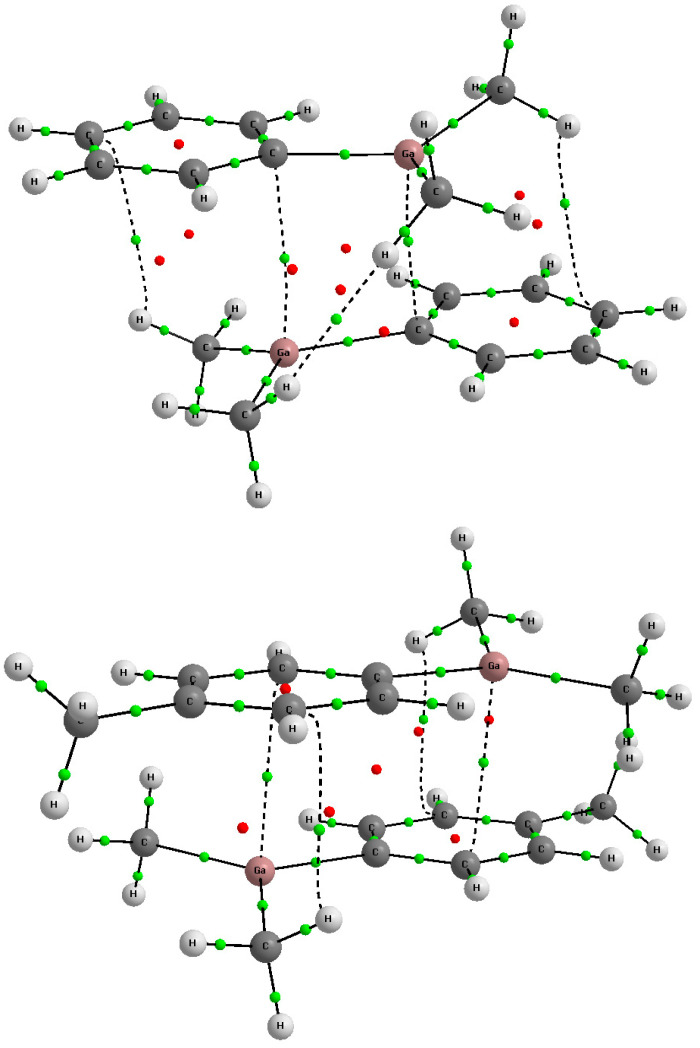
The molecular graphs of dimer structures of Dimethyl-phenyl-gallium (**up**), HOJBUE, and Dimethyl-(p-tolyl)-gallium (**down**), HOJCAL. The bond paths (solid and broken lines) and the critical points (BCPs—green, RCPs—red) are shown.

Table 3 presents various QTAIM parameters calculated for the shortest Ga···C contacts discussed earlier here. The parameters of bond critical points corresponding to these Ga···C bond paths are presented in the table as well as the QTAIM charges of the corresponding gallium and carbon centers being in contact. The delocalization parameter, δ(Ga,C), for these contacts is also included. [68,69] Figure 7 presents, as examples, molecular graphs [70,71] of two dimers theoretically analyzed here where stacking-like arrangements occur.

The electron density at the bond critical point, ρ_BCP_, is often treated as a measure of the strength of interaction since it often correlates with other parameters such as the interaction and binding energies or with the intermolecular distance. These correlations were analyzed for hydrogen bonds mainly [72,73,74] but they were also discussed for other types of interactions [67]. However, such relationships are observed if monomers in the complex are linked by single interactions. In other words, single short contacts are observed between monomers, such as in the trans-linear configuration of the water dimer [4] where the O-H···O hydrogen bond occurs and other atom–atom contacts are much longer and local interactions between them are negligible. In the case of stacking-like arrangements, in systems analyzed here, there are few shorter contacts between monomers which contribute to the total energy of interaction. Thus, QTAIM parameters of the single BCP located at the Ga···C bond path do not correlate with this energy. For example, it occurs for dimers presented in Figure 7 where aside from the Ga···C bond paths, C···H and even H···H bond paths are observed. These links (bond paths) correspond to local interactions thus consequently only correlations between local parameters may be observed here.

**Figure 8:**
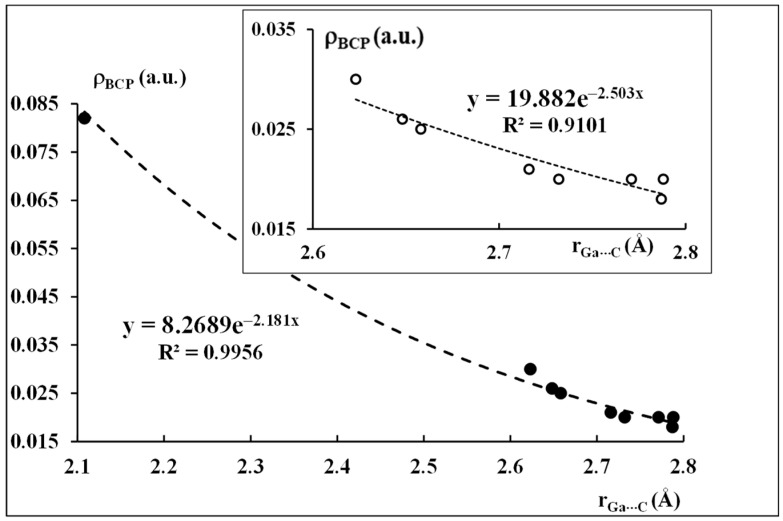
The exponential dependence between the Ga···C distance and the electron density at BCP at the corresponding bond path. The dependence on the right at the top presents the exponential relationship after the exclusion of the point corresponding to the shortest distance and the greatest ρ_BCP_ value.

Figure 8 is an example between local parameters since it presents the exponential dependence between the Ga···C distance and the electron density at the corresponding BCP, ρ_BCP_. The latter ρ_BCP_ parameter is related to the strength of the local interaction, thus the relationship of Figure 8 expresses character of local intermolecular forces which depend exponentially on the distance between interacting centers [67].

As it was pointed out here earlier, the above-mentioned local parameters are related to the shortest Ga···C contacts. One can see that one of the entries of this relationship is characterized by a very short Ga···C distance and a large corresponding ρ_BCP_ value. In other words, this entry is significantly different from the rest of entries, thus it should not be taken into account in this dependence from a statistical point of view. This is justified since this entry concerns the covalent in nature interaction (if not a covalent bond) in the dimethyl(cyclopentadienyl)gallium dimer (see Figure 4a), while other entries concern intermolecular contacts. If this “not matching” entry is excluded from the sample, the exponential relationship is much worse (R^2^ = 0.9101) and the second order polynomial dependence is much better (R^2^ = 0.9716). The dependence that is observed after the exclusion of the point mentioned above concerns the narrow range of distances (0.2 Å), in such cases of narrow ranges, the dependencies are often not well fitted to the exponential functions. However, it is obvious for the systems analyzed here that the increase of the ρ_BCP_ value is observed with the shortening of the Ga···C distance.

It is worth mentioning here that neither the Ga···C distance nor the corresponding ρ_BCP_ value correlates with the interaction energy; thus, it does not reflect the strength of interactions for systems analyzed here. However, two former parameters are local ones while the latter parameter concerns the whole dimer.

Let us discuss other local parameters collected in Table 3. The Laplacian of the electron density at BCP, ∇^2^ρ_BCP_ is positive in all cases, which may indicate no covalent bonds here. However, the total electron energy density, H_BCP_, is negative which is usually attributed in other studies to partially covalent in nature interactions [75,76,77,78]. In one case of the DMGACP structure for the above-discussed short Ga···C contact that may be classified as the covalent bond, the H_BCP_ value is “clearly negative” since it is equal to −0.037 au. For all remaining contacts, this value is very close to zero, thus it does not evidently indicate the partly covalent character.

One can see (Table 3) that there are no BCP characteristics for the OFUSAJ structure. This is because there are no short intermolecular contacts here and the bond paths are not detected. It was pointed out before here that this dimer is characterized by the weakest interaction (Table 2).

The QTAIM charges of the gallium center are situated in the +1.17–+1.20 au range. The negative QTAIM carbon charges of the centers in contact with the Ga center are characterized by the broader range, between −0.07 au and −0.42 au.

Table 3 also includes delocalization indices, δ(Ga,C) [68,69], for the Ga···C contacts analyzed in this study. The delocalization index may be treated as the measure of the covalent character of interaction (see Equation (1)).

δ(A,B) = −2 ∫_A,B_ (2Γ(r_1_,r_2_) − ρ(r_1_)ρ(r_2_))dr_1_dr_2_ (1)

ρ(r) and Γ(r_1_,r_2_) are one- and two-electron densities, respectively. Integrations are performed here through two atomic basins. The δ(A,B) index shows the number of electrons delocalized between two atoms (A and B). Hence it may be considered as the degree of covalency and as the number of shared electrons. One can see that this parameter refers to the local atom–atom contact (interaction), to the Ga···C contact in this study. Hence, it may correlate with other corresponding local parameters, for example, the linear correlation coefficient for the dependence between δ(Ga,C) and ρ_BCP_ is high (R^2^ = 0.997).

### 2.4. NBO Analysis

Table 4 presents NBO parameters of the theoretically analyzed dimers. The NBO atomic charges of Ga and C centers that are in contact are included. The range of the gallium center charge is much greater here than in the case of QTAIM charges since it is between +1.23 au and +1.35 au. Similarly, for the carbon NBO charge, the range is rather broad, between −0.26 au and −0.88 au. However, there are significant differences between definitions of QTAIM and NBO charges. For both definitions, the charges of nuclei are taken into account; however, for the QTAIM charges, the integration of the electron charge over the basins is considered while for NBO charges, the occupancies of orbitals are summarized.

Table 4 includes the Wiberg bond indices (WBIs) for the Ga···C contacts. The Wiberg index [79,80] corresponds to the bond order, it is related to the strength of the interatomic connection. The Wiberg Ga···C index correlates well with other characteristics related to these local atom–atom interactions. The linear correlation coefficients for relationships with the delocalization index, δ(Ga,C), and with the electron density at the Ga···C BCP, ρ_BCP_ are very high, R^2^ = 0.999 and 0.998, respectively.

Table 4 presents energies of orbital–orbital interactions for each Ga···C shortest contact of the dimer considered. For each contact, all overlaps are taken into account, thus the sum of energies of overlaps is presented. The following overlaps are observed for these contacts: n(C) → n(Ga), σ_CC_ → n(Ga), σ_CH_ → n(Ga), and π_CC_ → n(Ga). One can see that in all cases, for these overlaps, the unoccupied orbital, n(Ga), is the one of the gallium center that is characterized by the occurrence of π-hole. Therefore, the Ga···C local interactions are classified here as the π-hole triel bonds. In the case of DMGACP dimer, the Ga···C shortest contact corresponds to the 2.788 Å distance. For another Ga···C contact in the calculated dimer, the distance is equal to 2.108 Å, the NBO approach detects here the σ-bond orbital with polarization equal to 77.55%. The latter value means the percentage of the Ga-C σ-orbital electron density at the carbon center.

## 3. Materials and Methods 

Density functional theory, DFT, calculations were performed here with the use of the ADF2019 set of codes [81,82]. The BP86-D4/TZ2P level was applied for geometry optimizations and for frequency calculations on complexes corresponding to structures taken from CSD. They are characterized by links that may be classified as the Ga···C triel bonds; the detailed description of these complexes is given further here. The calculations do not show imaginary frequencies for them; thus, the optimized complexes correspond to energetic minima. For the level applied here, the BP86 functional [83,84] with the Grimme dispersion corrections [85] was used, and with the uncontracted Slater-type orbitals (STOs) as base functions with triple-ζ quality for all elements [86]. Relativistic scalar ZORA corrections [81] were applied for gallium atoms. The geometries optimized at the BP86-D4/TZ2P level were used further in other theoretical approaches.

The NBO approach [26,87] was applied to calculate the atomic charges, the energies of orbital–orbital interactions, and the Wiberg bond indices [79,80]. The NBO 6.0 program [88] implemented in the ADF2019 set of codes [81,82] was applied to perform NBO calculations.

The energy decomposition analysis, EDA [81,89], was applied here and the total interaction energies were partitioned according to the Equation (2).

ΔE_int_ = ΔE_elstat_ + ΔE_Pauli_ + ΔE_orb_ + ΔE_disp_ (2)

The ΔE_elstat_, ΔE_orb_, and ΔE_disp_ are attractive interaction energy terms corresponding, respectively, to the quasi-classical electrostatic interaction between the unperturbed charge distributions of atoms, to the charge transfer and polarization phenomena, i.e., to electron charge shifts resulting from the complex formation, and to the dispersion forces. The Pauli repulsion, ΔE_Pauli_, is the energy change associated with the transformation from the superposition of the unperturbed electron densities of the isolated fragments to the wave function that properly obeys the Pauli principle through antisymmetrization and renormalization of the product wave function. The convention is adopted that attractive energy terms are negative while the Pauli repulsion is positive in most studies.

The ADF2019 program [81,82] was also used to perform the quantum theory of atoms in molecules, QTAIM [90,91], calculations to analyze characteristics of the bond critical points, BCPs, corresponding to interactions that occur in complexes considered here. The characteristics of BCPs were calculated using the procedures of Rodriguez and co-workers [92,93,94] that are incorporated into the ADF2019 program. The delocalization indices [68,69] related to the intermolecular contacts were calculated in the same ADF QTAIM framework.

## 4. Conclusions

The CSD search was performed in this study to find crystal structures where trivalent gallium centers interact with the π-electron systems. Several structures were found but their analysis indicates that the gallium centers are not connected with π-electron sites rather but with the carbon centers. Hence, the Ga···C triel bonds are observed in these structures. This is partly in agreement with earlier theoretical studies [43,95]. In the case of boron and aluminum trihalides and trihydrides interacting with acetylene and ethylene, the connections of triel centers with π-electrons of CC bonds are observed [43] while in a case of interactions of these trihalides and trihydrides with benzene, B···C and Al···C links occur [95].

Most crystal structures analyzed here are characterized by the parallel (or nearly so) arrangements of molecules containing the gallium centers; they may be classified as stacking-like arrangements. In such structures, the intermolecular forces do not change the stable trigonal configurations, particularly into the tetrahedral ones. In the case of the crystal structure of dimethyl(cyclopentadienyl)gallium, molecules are linked by short Ga···C contacts, the cooperativity effects which enhance interactions are observed here. This is probably the first experimental evidence of the triel bonds´ cooperativity (other interactions do not cooperate in enhancing strengths of interactions in this structure).

The theoretical analyses confirm the experimental results. Mainly stacking-like dimers are observed as results of optimizations. The calculations show that the interactions of gallium species are electrostatic in nature but other attractive forces, orbital and dispersion interactions, also play an important role in stabilizing these systems. In general, the total interactions are rather strong and they consist of local atom–atom Ga···C, C···H, and H···H interactions. Therefore, despite strong total interactions in dimers, the weak local atom–atom interactions do not change the trigonal configurations of gallium centers. This is why the planar trigonal gallium structures are only slightly disturbed in crystal structures. One can say that the total strong interactions between monomers are “dispersed” into several weaker local atom–atom interactions, mainly attractive Ga···C interactions occur here. Similar stable planar structures of boron and aluminum trihalides and trihydrides were observed for their complexes with acetylene, ethylene, and benzene [43,95]. Only slight disturbances of trigonal structures occur sometimes [43]. It seems that it may be a common characteristic of stacking arrangements that the total interactions consist of several local atom–atom contacts. Other studies confirm that, for example, the early theoretical study on benzene and cytosine dimers [96].

## Figures and Tables

**Table 1:** Crystal structures resulting from CSD search that contain Ga···C triel bonds; refcodes, names of compounds, the shortest Ga···C distances (in Å) calculated (Calc) and taken from crystal structures (Cryst) are given. The references to original studies on these crystal structures are included (column Ref).

Refcode	Name	Cryst	Calc	Ref
DEDTUC	(μ_2_-1,4-Phenylenebis(3,3-dimethylbut-1-enyl))-tetraethyl-di-gallium	2.560	-	[44]
DMGACP	Dimethyl(cyclopentadienyl)gallium	2.314	2.788	[45]
HOJBUE	Dimethyl-phenyl-gallium	3.112	2.658	[46]
HOJCAL	Dimethyl-(p-tolyl)-gallium	3.117	2.787	[46]
HOJCEP	Dimethyl-(4-t-butylphenyl)-gallium	3.110	2.732	[46]
HOJCIT	Methyl-diphenyl-gallium	2.990	2.716	[46]
HUGVIP	(μ_2_-Benzene-1,4-diido)-tetramethyl-di-gallium	3.042	2.771	[47]
LEBNAH	Dimethyl-(N-methylpyrrol-2-yl)-gallium	2.481	2.623	[48]
NAZFIE	9,10-Dimethyl-9,10-dihydro-9,10-digalla-anthracene	2.696	2.648	[49]
NIZZUS	(5,10,15,20-Tetraphenylporphyrinato)-(trifluoromethanesulfonato)-gallium(iii) toluene solvate	3.439	-	[50]
OFUSAJ	Triethyl-gallium	3.087	2.923	[51]
QATMUT	(5,10,15-tris(Pentafluorophenyl)corrolato)-pyridine-gallium(iii) p-xylene solvate	3.430	-	[52]
UJIXEQ	1,12-Dimethyl-1,12-digalla(1.1)ferrocenophane	2.784	-	[53]

**Table 2:** The interaction energy and its terms (in kcal/mol) according to Equation (2) (see section Materials and Methods) for systems optimized here at the BP86-D4/TZ2P level; dimers calculated correspond to crystal structures (their refcodes are given in the first left column). The percentage contribution of the electrostatic energy to the sum of all attractive contributions is given, %ΔE_elstat_.

Refcode	ΔE_int_	ΔE_Pauli_	ΔE_elstat_	ΔE_orb_	ΔE_disp_	%ΔE_elstat_
DMGACP	−11.90	24.12	−14.81	−10.94	−10.27	41.1
HOJBUE	−27.23	60.01	−37.02	−29.49	−20.73	42.4
HOJCAL	−24.05	43.40	−25.26	−20.98	−21.20	37.5
HOJCEP	−26.74	51.04	−29.07	−24.68	−24.04	37.4
HOJCIT	−25.73	51.20	−29.18	−24.54	−23.21	37.9
HUGVIP	−27.90	50.65	−28.76	−24.75	−25.04	36.6
LEBNAH	−37.10	85.91	−57.17	−45.29	−20.55	46.5
NAZFIE	−58.25	145.83	−89.93	−70.29	−43.86	44.1
OFUSAJ	−11.77	24.00	−10.98	−12.53	−12.26	30.7

**Table 3:** The QTAIM parameters (in au) of the shortest Ga···C contacts of dimers calculated at the BP86-D4/TZ2P level. The following parameters are included; the electron density at BCP, ρ_BCP_, the Laplacian of electron density at BCP, ∇^2^ρ_BCP_, the total electron energy density at BCP, H_BCP_, the charges of Ga and C centers being in the contact, qGa and qC, respectively, and the delocalization parameter of Ga···C contact, δ(Ga,C).

Refcode	ρ_BCP_	∇^2^ρ_BCP_	H_BCP_	qGa	qC	δ(Ga,C)
DMGACP	0.020	0.036	−0.001	1.165	−0.095	0.116
DMGACP	0.082	0.096	−0.037	1.165	−0.261	0.492
HOJBUE	0.025	0.046	−0.002	1.186	−0.409	0.149
HOJCAL	0.018	0.038	−0.001	1.194	−0.067	0.103
HOJCEP	0.020	0.041	−0.001	1.197	−0.088	0.114
HOJCIT	0.021	0.042	−0.001	1.193	−0.082	0.117
HUGVIP	0.020	0.039	−0.001	1.190	−0.068	0.105
LEBNAH	0.030	0.050	−0.004	1.202	−0.107	0.161
NAZFIE	0.026	0.050	−0.002	1.202	−0.419	0.141
OFUSAJ				1.166		

**Table 4:** The NBO charges of Ga and C centers being in the shortest contact, WBI—Wiberg Ga···C bond index, E_NBO_—the sum of orbital–orbital interaction energies (in kcal/mol).

Refcode	qGa	qC	WBI	E_NBO_
DMGACP	1.285	−0.345	0.092	23.4
DMGACP	1.285	−0.545	0.438	77.55 *
HOJBUE	1.289	−0.488	0.125	27.69
HOJCAL	1.307	−0.258	0.078	16.42
HOJCEP	1.298	−0.266	0.088	14.45
HOJCIT	1.317	−0.278	0.092	17.79
HUGVIP	1.299	−0.268	0.082	19.54
LEBNAH	1.227	−0.337	0.144	29.30
NAZFIE	1.351	−0.497	0.111	16.48
OFUSAJ	1.289	−0.875	0.061	11.95

* This is the polarization of the Ga–C bond.

## Data Availability

Not applicable.
